# Sodium-glucose Cotransporter-2 Inhibitors and Euglycaemic Diabetic Ketoacidosis in the Perioperative Period: Case Report

**DOI:** 10.7759/cureus.5455

**Published:** 2019-08-21

**Authors:** Urmita Jhaveri, Deepak Vardesh

**Affiliations:** 1 Medicine, Logan Hospital, Brisbane, AUS; 2 Surgery, Logan Hospital, Brisbane, AUS

**Keywords:** sglt2 inhibitors, euglycaemic ketoacidosis, perioperative, empagliflozin

## Abstract

The use of sodium-glucose cotransporter-2 inhibitors (SGLT2i) has been steadily increasing over the past few years due to their efficacy in glycaemic control as well as added benefits of weight loss and reduction in cardiovascular mortality. SGLT2i are a class of oral hypoglycaemics that work by increasing urinary glucose excretion via the inhibition of the sodium-glucose cotransporter-2 in the proximal tubule of the kidney. Euglycaemic diabetic ketoacidosis (euDKA) is a potentially life-threatening adverse effect of SGLT2i. The literature shows an increasing awareness of this issue amongst physicians. However, in addition to prescriber education, emphasis needs to be placed on patient education to highlight this potentially serious adverse effect. We present two cases of patients with euDKA following SGLT2i use in the perioperative period. The cases discussed reiterate the importance of awareness of SGLT2i-induced euDKA during the perioperative period. Both cases raise the question of whether patients are being adequately educated about the drug, its adverse effects and under what conditions to cease the medication.

## Introduction

The use of sodium-glucose cotransporter-2 inhibitors (SGLT2i) has been steadily increasing over the past few years due to their efficacy in glycaemic control as well as added benefits of weight loss and reduction in cardiovascular mortality [1-3]. Euglycaemic diabetic ketoacidosis (euDKA) is an underrecognised, potentially life-threatening adverse effect of SGLT2i and is a diagnostic challenge [4, 5]. The literature shows an increasing awareness of this issue amongst anaesthetists, endocrinologists and perioperative physicians [6, 7]. However, in addition to prescriber education, emphasis needs to be placed on patient education to increase awareness of this potentially serious adverse effect. We discuss two cases of euDKA in perioperative patients on SGLT2i and review existing literature.

## Case presentation

Case 1

A 43-year-old obese female with a five-year history of type 2 diabetes mellitus presented with acute cholecystitis. She had presented four days earlier with abdominal pain and had been diagnosed with cholelithiasis and advised elective cholecystectomy. Her past medical history included hypertension and hypothyroidism. She had received insulin and metformin therapy in the past. She was intolerant of metformin. Her insulin had subsequently been ceased and she was commenced on empagliflozin instead. While unwell, she had been vomiting with minimal oral intake but had continued to take all her medications including empagliflozin.

She underwent emergency laparoscopic cholecystectomy. Intra-operatively she was noted to be profoundly acidotic on an arterial blood gas with a pH of 6.82 (7.35-7.45), bicarbonate of 6 mmol/L (22-26 mmol/L), anion gap of 28 mmol/L (8-16 mmol/L) and glucose of 19.1 mmol/L (4.0-7.8 mmol/L). She was subsequently transferred to the intensive care unit (ICU) for post-operative management where she was commenced on an insulin infusion. Her acidosis and ketosis gradually resolved over the next 36 hours. In view of the acidosis being out of proportion to her hyperglycaemia and her being on an SGLT2 inhibitor, she was diagnosed with severe SGLT2i-euDKA. Her SGLT2i was discontinued and she was discharged home on regular long-acting insulin (insulin glargine) therapy.

Case 2

A 41-year-old female presented seven days post-left groin lymph node biopsy with a three-day history of worsening pain of her surgical wound with associated erythema and purulent discharge. She remained systemically well with good oral intake. Ultrasound of her groin confirmed an abscess and she underwent an incision and drainage the following day. Her other active medical issues included poorly-controlled type 2 diabetes mellitus (HbA1c 11.7%, target range 6.5%-7.5%) on oral hypoglycaemics, dyslipidaemia, recurrent episodes of supraventricular tachycardia, obesity, cutaneous T-cell lymphoma and mild obstructive sleep apnoea. Her medications on admission included metformin-empagliflozin 1000 mg-12.5 mg twice a day, fenofibrate 96 mg once daily and celestone 0.02% topical cream as required. She continued to take her metformin-empagliflozin combination at home until the night before presentation, however, it was appropriately withheld at the time of presentation.

Despite this, she suffered from post-operative euDKA with a pH of 7.29, bicarbonate of 14 mmol/L, anion gap of 18 and glucose of 13.9 mmol/L. She remained haemodynamically stable and cares were managed on the surgical ward with physician input. She was commenced on an insulin infusion which was continued for four days. Once her ketoacidosis resolved, she was recommenced on metformin and discharged on insulin as per recommendations made by the endocrinologist. The patient was hesitant to stop her empagliflozin and commence insulin due to concerns about weight gain. However, after a lengthy discussion about the risks of SGLT2i-induced euDKA, she agreed to continue insulin indefinitely and only to retrial empagliflozin/other SGLT2i in the future under close supervision of an endocrinologist.

## Discussion

SGLT2i are a class of oral hypoglycaemics prescribed for type 2 diabetes mellitus that work by increasing urinary glucose excretion. They do so by inhibiting the sodium-glucose cotransporter-2 in the proximal tubule of the kidney. This cotransporter is responsible for glucose resorption and thus, its inhibition increases glycosuria and decreases serum glucose levels [1]. The recent increase in use of SGLT2i may be in light of their many added benefits including weight loss, reduced systolic blood pressure, reduced hospitalisations from decompensated heart failure, reduced cardiovascular mortality and reno-protection [2, 3, 8, 9]. The Australian Pharmaceutical Benefits Scheme (PBS) subsidises the use of two SGLT2i, dapagliflozin and empagliflozin (Table 1), as stand-alone medications or in combination with metformin [10-12].

**Table 1:** Comparison of SGLT2i available on Australian PBS PBS: Pharmaceutical Benefits Scheme

Generic Name	Brand Name	Other Formulation	Average Plasma Half-life
Empagliflozin	Jardiance	Empagliflozin/metformin (Jardiamet)	12.4 hours
Dapagliflozin	Forxiga	Dapagliflozin/metformin (Xigduo XR)	12.9 hours

Diabetic ketoacidosis is a medical emergency defined as a triad of hyperglycaemia >14 mmol/L, elevated plasma ketones and metabolic acidosis [5]. Euglycaemic DKA is termed as such due to the level of hyperglycaemia not being as profound as expected in DKA. This mild hyperglycaemia may be the reason for the delay in diagnosis of this rare condition [13]. The hypothesised pathogenesis behind SGTL2i-associated euDKA is a combination of relative insulin deficiency, glucagon excess and a shift towards fatty acid metabolism [9, 13]. SGLT2i-induced glycosuria reduces plasma glucose concentration which decreases insulin secretion. In addition to this, inhibition of SGLT2 transporters present in pancreatic alpha cells triggers the release of glucagon. These alterations of circulating glucagon and insulin levels predispose individuals to ketosis by causing a shift towards lipolysis [13]. The perioperative period, which often includes patients with active infection, periods of fasting and temporary reduction or cessation of insulin therapy, can predispose patients on SGLT2i to euDKA. Other triggers and predisposing factors include latent autoimmune diabetes of adulthood, undiagnosed type 1 diabetes mellitus, extensive exercise, alcohol and corticosteroid use. A systematic review published by Burke et al. reported that 28% of euDKA cases were precipitated by major surgery [7]. Following several case reports of this adverse effect, the Australian Diabetes Society (ADS) and the Australian and New Zealand College of Anaesthetists (ANZCA) issued alerts about severe euDKA with SGLT2i use in the perioperative period [14, 15]. A summary of these recommendations is listed in Table 2.

**Table 2:** Summary of recommendations made by the ADS and ANZCA. ADS: Australian Diabetes Society; ANZCA: Australian and New Zealand College of Anaesthetists.

Period	Recommendations
Preoperative Period	Cease SGLT2i use three days pre-operatively or in other physically stressful situations. Strongly consider postponing non-urgent surgical procedures if SGLT2i have not been ceased three days prior to surgery and blood ketones are >0.6 mmol/L, or HbA1c >9.0%.
Perioperative Period	Routinely check blood glucose and ketone levels. If the blood ketone level is >0.6 mmol/L in pre- or perioperative patients, an urgent arterial or venous blood gas must be performed. EuDKA should be treated as a medical emergency. All patients with euDKA should be reviewed by an endocrinologist or physician on-call.
Postoperative Period	Withhold SGLT2i until the patient has returned to a full diet in case of day procedures, or until the patient is eating and drinking well and close to discharge (3-5 days post-operatively) in cases of major surgery.

The cases described here highlight the importance of awareness of SGLT2i-induced euDKA during the perioperative period. Despite the first patient having poor oral intake at home, she continued to take her SGLT2i and was subsequently admitted to ICU post-operatively. It is important to note that she presented four days prior with similar symptoms and was discharged with the plan for an elective cholecystectomy. During that first presentation, the patient was not appropriately advised on when to cease use of her SGLT2i. This raises the question of whether patients are being adequately educated about the drug, its adverse effects and under what conditions to cease this medication. In our second case, the SGLT2i was ceased appropriately at presentation by medical officers, however not by the patient whilst she was suffering with an active infection. Furthermore, the second case highlights the importance of being vigilant about SGLT2i-induced euDKA given the patient suffered from this rare complication whilst being systemically quite well and undergoing a minor surgery.

## Conclusions

Euglycaemic DKA is a rare but increasingly recognised adverse effect of SGLT2i during the perioperative period. While physicians and anaesthetists are becoming more aware of this issue, emphasis needs to be placed on patient education, so they are aware to stop this medication at least three days prior to surgery or in instances of physiological stress. Additionally, health professionals must have a high index of suspicion for euDKA in acutely unwell patients using SGLT2i.

## References

[1] Taylor SI, Blau JE, Rother KI (2015). SGLT2 inhibitors may predispose to ketoacidosis. J Clin Endocrinol Metab.

[2] Zinman B, Wanner C, Lachin JM (2015). Empagliflozin, cardiovascular outcomes, and mortality in type 2 diabetes. N Engl J Med.

[3] Mosley JF II, Smith L, Everton E, Fellner C (2015). Sodium-glucose linked transporter 2 (SGLT2) inhibitors in the management of type-2 diabetes: a drug class overview. P T.

[4] Peters AL, Buschur EO, Buse JB, Cohan P, Diner JC, Hirsch IB (2015). Euglycemic diabetic ketoacidosis: a potential complication of treatment with sodium-glucose cotransporter 2 inhibition. Diabetes Care.

[5] Chacko B, Whitley M, Beckmann U, Murray K, Rowley M (2018). Postoperative euglycaemic diabetic ketoacidosis associated with sodium-glucose cotransporter-2 inhibitors (gliflozins): a report of two cases and review of the literature. Anaesth Intensive Care.

[6] Goldenberg RM, Berard LD, Cheng AYY, Gilbert JD, Verma S, Woo VC, Yale JF (2016). SGLT2 inhibitor-associated diabetic ketoacidosis: clinical review and recommendations for prevention and diagnosis. Clin Ther.

[7] Burke KR, Schumacher CA, Harpe SE (2017). SGLT2 inhibitors: a systematic review of diabetic ketoacidosis and related risk factors in the primary literature. Pharmacotherapy.

[8] Heerspink HJL, Desai M, Jardine M, Balis D, Meininger G, Perkovic V (2017). Canagliflozin slows progression of renal function decline independently of glycemic effects. J Am Soc Nephrol.

[9] Qiu H, Novikov A, Vallon V (2017). Ketosis and diabetic ketoacidosis in response to SGLT2 inhibitors: basic mechanisms and therapeutic perspectives. Diabetes Metab Res Rev.

[10] (2019). SGLT2 inhibitor listings: indications and combinations. https://www.nps.org.au/radar/articles/sglt2-inhibitor-listings-indications-and-combinations.

[11] Pharmaceuticals BI (2019). Full prescribing information for JARDIANCE. https://docs.boehringer-ingelheim.com/Prescribing%20Information/PIs/Jardiance/jardiance.pdf.

[12] Pharmaceutical A (2019). Full prescribing information FARXIGA. https://www.azpicentral.com/farxiga/farxiga.pdf#page=1.

[13] Palmer BF, Clegg DJ, Taylor SI, Weir MR (2016). Diabetic ketoacidosis, sodium glucose transporter-2 inhibitors and the kidney. J Diabetes Complications.

[14] Society AD (2019). Severe euglycaemic ketoacidosis with SGLT2 inhibitor use in the perioperative period. https://diabetessociety.com.au/documents/2018_ALERT-ADS_SGLT2i_PerioperativeKetoacidosis_v3__final2018_02_14.pdf.

[15] Traill DR (2019). Severe euglycaemic ketoacidosis with SGLT2 inhibitor use in the perioperative period. http://www.anzca.edu.au/documents/alert-dka-and-oral-hypoglycaemics-20180215.pdf.

